# An exosome mRNA-related gene risk model to evaluate the tumor microenvironment and predict prognosis in hepatocellular carcinoma

**DOI:** 10.1186/s12920-024-01865-z

**Published:** 2024-04-16

**Authors:** Zhonghai Du, Xiuchen Han, Liping Zhu, Li Li, Leandro Castellano, Justin Stebbing, Ling Peng, Zhiqiang Wang

**Affiliations:** 1 Department of Medical Oncology, Weifang Hospital of Traditional Chinese Medicine, Weifang, Shandong Province China; 2 Department of Surgical Oncology, National Cancer Center/National Clinical Research Center for Cancer/Cancer Hospital and Shenzhen Hospital, Chinese Academy of Medical Sciences and Peking Union Medical College, Shenzhen, 518116 China; 3 Department of Medical Oncology, Shouguang Hospital of Traditional Chinese Medicine, Shouguang, Shandong Province China; 4 Outpatient Surgery Center, Shouguang Hospital of Traditional Chinese Medicine, Shouguang, Shandong Province China; 5 Department of Biochemistry, School of Life Sciences, University of Sussex, Brighton, United Kingdom; 6 Department of Surgery and Cancer, Division of Cancer, Imperial College London, London, United Kingdom; 7 Department of Life Sciences, Anglia Ruskin University, Cambridge, United Kingdom; 8 Department of Pulmonary and Critical Care Medicine, Cancer Center, Zhejiang Provincial People’s Hospital, Affiliated People’s Hospital, Hangzhou Medical College, Hangzhou, Zhejiang China; 9 Department of Urology, Shouguang Hospital of Traditional Chinese Medicine, Shouguang, Shandong Province China

**Keywords:** Hepatocellular carcinoma, Extracellular vesicle, Exosome, Risk score, Prognostic signature, Tumor immune microenvironment, PRKDC

## Abstract

**Background:**

The interplay between exosomes and the tumor microenvironment (TME) remains unclear. We investigated the influence of exosomes on the TME in hepatocellular carcinoma (HCC), focusing on their mRNA expression profile.

**Methods:**

mRNA expression profiles of exosomes were obtained from exoRBase. RNA sequencing data from HCC patients’ tumors were acquired from The Cancer Genome Atlas (TCGA) and the International Cancer Genome Consortium (ICGC). An exosome mRNA-related risk score model of prognostic value was established. The patients in the two databases were divided into high- and low-risk groups based on the median risk score value, and used to validate one another. Functional enrichment analysis was performed based on a differential gene prognosis model (DGPM). CIBERSORT was used to assess the abundance of immune cells in the TME. The correlation between the expression levels of immune checkpoint-related genes and DGPM was analyzed alongside the prediction value to drug sensitivity.

**Results:**

A prognostic exosome mRNA-related 4-gene signature (DYNC1H1, PRKDC, CCDC88A, and ADAMTS5) was constructed and validated. A prognostic nomogram had prognostic ability for HCC. The genes for this model are involved in extracellular matrix, extracellular matrix (ECM)-receptor interaction, and the PI3K-Akt signaling pathway. Expression of genes here had a positive correlation with immune cell infiltration in the TME.

**Conclusions:**

Our study results demonstrate that an exosome mRNA-related risk model can be established in HCC, highlighting the functional significance of the molecules in prognosis and risk stratification.

**Supplementary Information:**

The online version contains supplementary material available at 10.1186/s12920-024-01865-z.

## Introduction

Hepatocellular carcinoma (HCC) is one of the most fatal cancers worldwide [1]. In the past 20 years, treatment options of HCC have been primarily chemotherapy, radiotherapy, and surgery. Recently, anti-angiogenic drugs and immune checkpoint inhibitors (ICIs) have demonstrated promise here [2]. However, a large number of HCC patients inevitably experience relapse or disease progression after initial treatment [3]. To identify high-risk patients with a poor prognosis and inform treatment decisions are of vital importance in HCC.

Extracellular vesicles (EVs) are defined as lipid bilayer packages of biological materials, released from various type of cells into surrounding environment. EVs have been considered as ideal biomarkers for the diagnosis and prognosis of cancer [4]. EVs include particles such as exosomes, microvesicles, ectosomes, oncosomes, and apoptotic bodies. Among the main types of EVs, exosomes contain large amounts of RNAs, which can be transmitted among cells and modulate the gene expression of recipient cells [5]. Exosomes contain messenger RNA (mRNA), circular RNA (circRNA), long non-coding RNA (lncRNA), microRNA (miRNA), lipids, and proteins [6]. As intercellular messengers between cells, exosomes can regulate cell differentiation and tissue development [7]. Exosomal RNAs can interact with many types of cancers and are associated with several hallmarks features of cancer. The liquid biopsy approach of exosomes has been used as tumor markers [8]. Based on the ability of exosomes to carry biomolecules to different tissues, exosomes also have application potential in cancer therapy [9]. The potential of using exosomes to predict response to immunotherapy has also been investigated [10].

Exosomes play a vital role in the development, progression, and metastases of HCC [11]. Previous studies confirmed that exosomes could promote progression and metastasis of HCC by regulating multiple signaling pathways and modulating the TME [12]. Furthermore, as exosomes release inhibitory and stimulatory contents that facilitate the cross-talk of tumor cells and the TME, exosomes demonstrate potential for overcoming resistance mechanisms of anti-cancer drugs.

In this study, we aimed to explore potential functional mRNAs in progression and development of HCC and the immune microenvironment of HCC. The study results highlight that exosomal mRNAs which could act as prognostic biomarkers for HCC.

## Materials and methods

### The mRNA expression data collection

The exoRBase database (http://exorbase.org/exoRBaseV2/download/toIndex) is a depository of mRNAs, lncRNAs, and circRNAs from RNA sequencing (RNA-seq) data analyses in different human body fluids [13]. ExoRBase provides expression landscapes and a comprehensive annotation of extracellular vesicle long RNAs (exLRs), which will help discover novel exLR signatures and facilitate the identification of new circulating biomarkers for cancer therapy. In the current study, the mRNA expression profiles were obtained from the exoRBase database up to April 30, 2023, which included 112 HCC, 130 benign tumor and 118 healthy blood samples.

### TCGA-LIHC cohort and ICGC (LIRI-JP) cohort

A total of 374 HCC patients from the TCGA-LIHC cohort were identified. Among them, the level 3 RNA-seq data, somatic mutations data and clinical data of 371 HCC patients with complete information were retrieved from the TCGA website (https://portal.gdc.cancer.gov/projects/TCGA-LIHC/). RNA-seq data, somatic mutations data and clinical information of 231 tumor samples (LIRI-JP cohort) from the ICGC database were downloaded from ICGC portal (https://dcc.icgc.org/projects/LIRI-JP). The samples from LIRI-JP cohort were primarily derived from Japanese HCC patients with HBV or HCV infection.

### Differentially expressed mutant genes (DEMGs)

The 118 healthy blood samples were used as the normal group. The differentially expressed genes (DEGs) in blood samples from 130 benign and 112 HCC patients were identified of *p* < 0.05 and fold-change (FC) > 0 via “limma” R package, respectively. Integration analyses between normal vs. HCC and normal vs. benign comparisons were performed. Those mRNAs that were only significantly differentially expressed in normal vs. HCC comparison (not in normal vs. benign comparison) were screened out. Then, the DEMGs of HCC were obtained from the intersection genes that have mutations in TCGA, ICGC, and exoRBase.

### Construction and validation of a differential gene prognosis model (DGPM)

The DEGs between tumor and adjacent tissues were identified with a false discovery rate (FDR) < 0.05 in the TCGA cohort. Univariate Cox analysis was carried out to screen DEMGs with prognostic values in terms of overall survival (OS). *P* values were adjusted by Benjamini & Hochberg (BH) correction. Visualization and comparison of gene alterations were conducted with cBioPortal. LASSO-penalized Cox regression analysis was used to construct a DGPM to minimize the risk of overfitting. LASSO algorithm was used for to select variables with “glmnet” R package. Penalty parameter (λ) was chosen by 10-fold cross-validation with the minimum criteria. The risk scores were calculated with the normalized expression level of each gene and corresponding regression coefficients. The risk score was calculated with the following formula:

$$The\;risk\;score=\sum_{i=1}^n{Coef}_i\times{Expr}_i$$

where Expr*i* indicates the expression level of gene *i,* and coef*i* means the regression coefficient of gene *i*.

The patients were separated into high- and low-risk groups due to the median value of the risk score. Principal component analysis (PCA) was performed with “stats” R package. Besides, t-distributed stochastic neighbor embedding (t-SNE) was used to investigate the distribution of different groups using “Rtsne” R package. The optimal cut-off value for gene expression was determined by “survminer” R package. The time-dependent receiver operating characteristic (ROC) curve analyses were conducted with “survivalROC” R package. Survival analysis was performed to validate the prognostic performance independent from clinical parameters. For the validation studies, LIRI-JP cohort was employed. The risk score was calculated with the same formula used with TCGA cohort. The patients in the ICGC cohort were also divided into low- or high-risk subgroups by applying the median risk score from TCGA cohort, and these groups were then compared to confirm the gene model.

### Functional enrichment analysis

Gene Ontology (GO) and Kyoto Encyclopedia of Genes and Genomes (KEGG) analyses were performed with “clusterProfiler” R package based on the DEMGs between high- and low-risk groups. The infiltrating score of immune cells and the activity of immune-related pathways were analysed with single-sample gene set enrichment analysis (ssGSEA) with “gsva” R package.

### Construction of the prognostic nomogram

To comprehensively assess prognosis predictive ability of risk signature, tumor stage, gender, age, WHO grade, T category, N category and M category for 1-, 3-, and 5-year OS, time-dependent ROC curves was performed to calculate the area under the curve (AUC) values. Prognostic nomogram which containing DEMG-based risk model and clinical parameters was established.

### Correlation of risk score with immune cells

The information for immune infiltration was downloaded from the tumor immune estimation resource (TIMER) (https://cistrome.shinyapps.io/timer/). A correlation between prognostic risk score and immune cell infiltration was performed. SsGSEA was performed to investigate the enrichment of the two subgroups in immune function-associated gene sets via “GSEAbase” R package. “ESTIMATE” R package was used to evaluate tumor purity and infiltrating cells, including immune cell and stromal cell. The fraction of 22 immune cell types was assessed by cell type identification by estimating relative subsets of RNA transcripts (CIBERSORT; https://cibersort.stanford.edu/).

### Role of risk score in immune checkpoint blockade (ICB) treatment

Herein, 6 key genes of ICB treatment in HCC were extracted, including cytotoxic T-lymphocyte antigen 4 (CTLA-4), programmed death 1 (PD-1, or PDCD1), programmed death ligand 1 (PD-L1, or CD274), programmed death ligand 2 (PD-L2, or PDCD1LG2), T-cell immunoglobulin domain and mucin domain-containing molecule-3 (TIM-3, or HAVCR2), and indoleamine 2,3-dioxygenase 1 (IDO1). DEMG-based prognostic signature and expression level of 6 ICB key genes were correlated. Furthermore, the expression level of 47 ICB-related genes between low- and high-risk groups were also compared.

### Statistical analysis

All statistical analyses were conducted by R software (version 4.1.1). Gene expression was analysed by student’s t-test. Differences in proportions were analysed with Chi-squared test. ssGSEA of immune cells or pathways between the groups were examined with Mann-Whitney test. Kaplan-Meier curves were employed to assess survival data. Pearson correlation test was used to analyze the correlation of risk score, clinical parameters, immune cell infiltration, and immune checkpoints. Independent prognostic performance of risk signature was evaluated with Cox regression models. *p* value < 0.05 was considered as statistically significant.

## Results

### Identification of prognostic related DEMGs from the exoRBase database

**Fig. 1:**
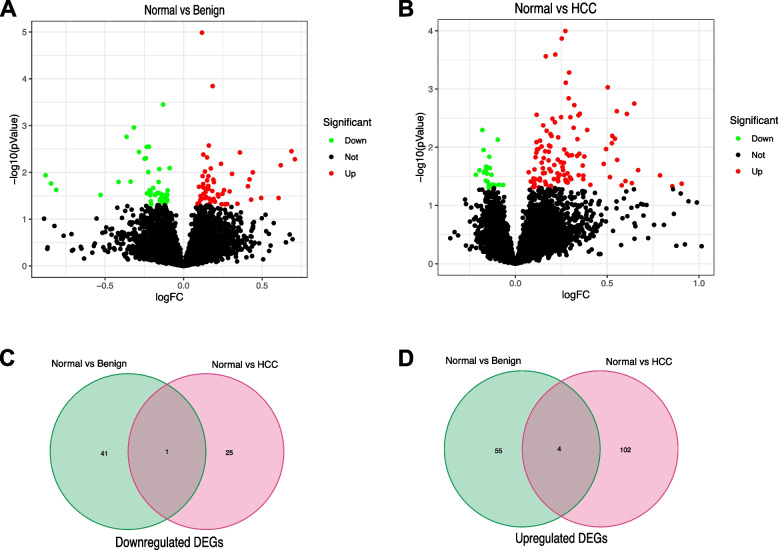
Candidate mRNAs in HCC. **A** Volcano plot of DEGs between 130 benign and 118 healthy blood samples of exosome. **B** Volcano plot of DEGs between 112 HCC and 118 healthy blood samples of exosome. **C** Interaction analysis of downregulated (**C**) and upregulated (**D**) DEGs in both compared groups

To explore potential mRNAs associated with development and progression of HCC, the blood samples of 112 HCC, 130 benign and 118 healthy people from the exoRBase database were investigated, after differential expression analysis between normal and benign or blood samples of HCC patients. Compared with normal blood samples, 59 and 42 mRNAs were significantly upregulated and downregulated in benign blood samples, respectively (Fig. 1A and Supplementary Table 1). In addition, a total of 132 significant DEGs (106 upregulated and 26 downregulated ones) were identified in normal vs. HCC (Fig. 1B and Supplementary Table 2). Integration analyses between normal vs. HCC and normal vs. benign were performed. The mRNAs only significantly differentially expressed in normal vs. HCC (not in normal vs. benign) were screened out. Consequently, 102 upregulated and 25 downregulated mRNAs were identified (Fig. 1C-D).

**Fig. 2:**
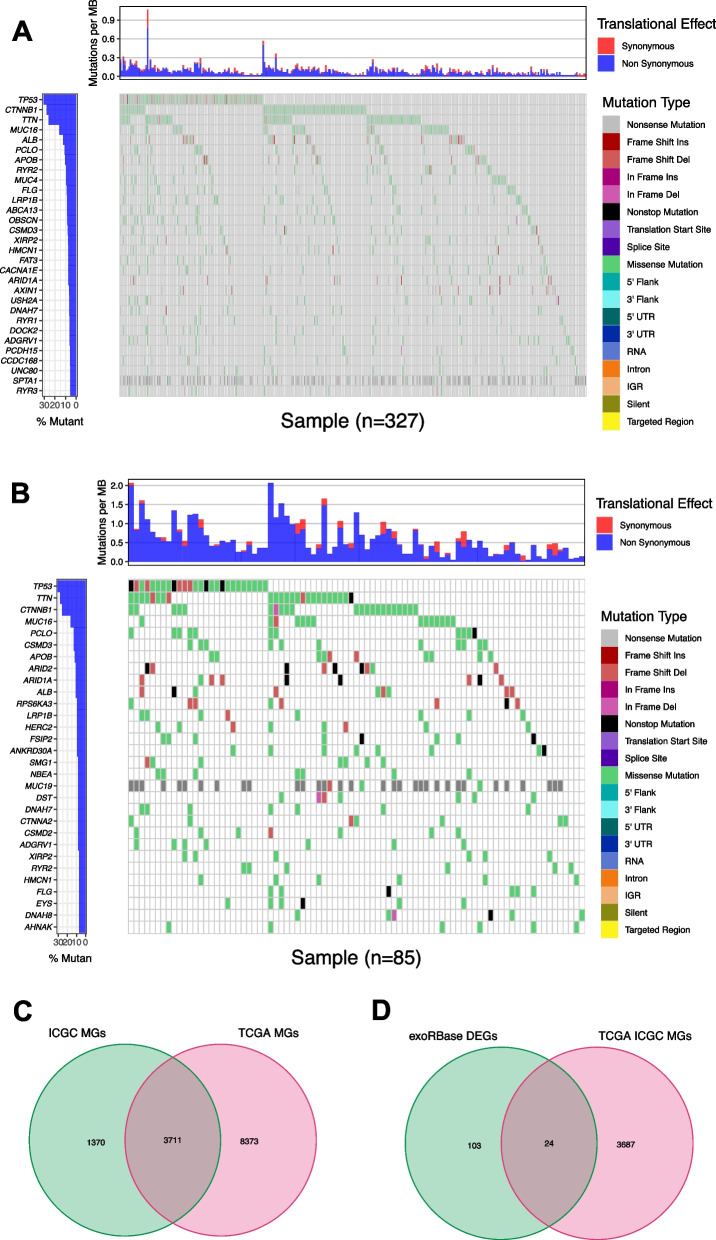
Frequently mutated genes in HCC. **A** The frequently mutated genes in HCC from TCGA cohort were depicted with Oncoplot. **B** The frequently mutated genes in HCC from ICGC cohort were displayed by waterfall plot. **C** Venn diagram of genes covered by both TCGA and ICGC cohorts. **D** Interaction analysis for DEMGs in both compared groups

In addition, 374 HCC patients from TCGA-LIHC cohort and 273 HCC patients from ICGC cohort were enrolled. The frequently mutated genes in American HCC samples from TCGA cohort (Fig. 2A) and ICGC cohort (Fig. 2B) were identified. Of note, there were some frequently mutated genes in both American and Japanese patients. Comparative analysis of mutated genes between TCGA and ICGC cohorts were performed (Fig. 2C). Then, we analyzed the DEGs from the exoRBase database, and 24 HCC DEMGs were obtained (Fig. 2D).

**Fig. 3:**
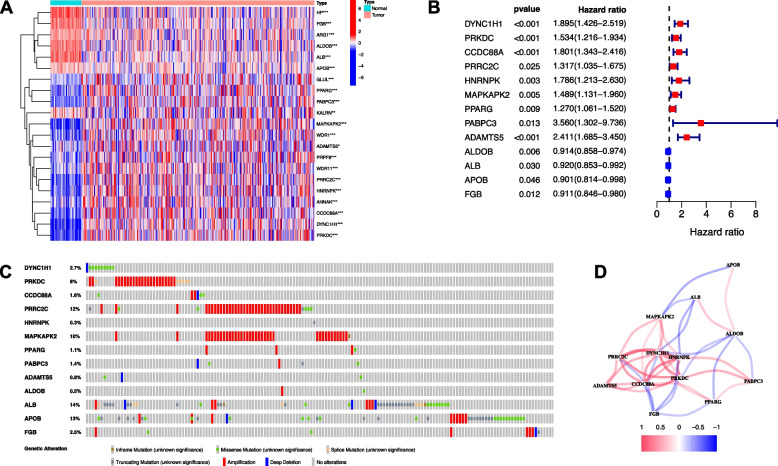
Candidate prognostic related DEMGs in the TCGA cohort. **A** Heatmap of the prognostic related DEMGs between normal and tumour tissues. **B** Forest plots showing the results of the univariate Cox regression analysis between gene expression and OS. **C** Landscape of prognostic related DEMGs alteration in HCC. **D** The correlation network of candidate genes

The expression levels of 24 DEMGs were compared in the pooled TCGA and Genotype-Tissue Expression (GTEx) data from 50 normal and 374 tumor tissues, and 13 DEMGs were identified (Fig. 3A). Among them, 4 genes (APOB, FGB, ALDOB, and ALB) were downregulated while 9 other genes (PPARG, PABPC3, MAPKAPK2, ADAMTS5, CCDC88A, PRRC2C, HNRNPK, DYNC1H1, and PRKDC) were enriched in the tumour group. All of the 13 genes were associated with OS in the univariate Cox regression analysis (Fig. 3B). Mutations of the 13 genes were analyzed by cBioPortal (Fig. 3C). The correlation of the genes is presented in Fig. 3D.

### Development of a prognostic gene model in TCGA cohort

**Fig. 4:**
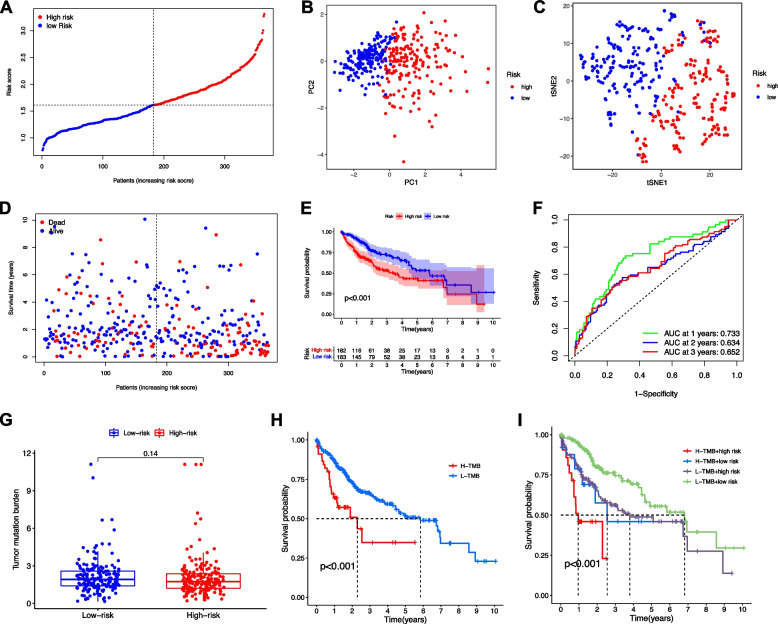
Prognostic value of the risk score in the TCGA cohort. **A** Distribution and median value of the risk scores. **B** PCA plot and **C** t-SNE analysis. **D** Distributions of OS status, OS and risk score. **E** Kaplan-Meier curves for the OS of patients in the high- and low-risk group. **F** AUC of time-dependent ROC curves confirmed the prognostic performance of the risk score. **G** Comparison of TMB between low- and high-risk groups. **H** Survival analysis based on the TMB. **I** Survival analysis for 4 groups by combining TMB and DEMG-based risk signature

A prognostic model was established by LASSO Cox regression with the expression profile of the 13 genes identified above. As a result, the 4-gene signature based on the optimal value of λ was identified (DYNC1H1, PRKDC, CCDC88A, and ADAMTS5, Table 1). Survival analyses suggested that high expression of these genes correlated with a poor prognosis according to the optimal cut-off expression value of each gene (all adjusted *p* < 0.05). The patients were stratified into high- and low-risk group based on the median cut-off value (Fig. 4A). The patients in different risk groups were distributed in two directions as PCA and t-SNE analysis indicated (Fig. 4B-C). Patients in high-risk group had a higher probability of death earlier (Fig. 4D) and a significantly worse OS (Fig. 4E). The AUCs reached 0.733, 0.634, and 0.652 at 1-, 3-, and 5-year, respectively (Fig. 4F).

**Table 1:** Genes selected to construct the prognostic model

Gene	Control mean	Treatment mean	logFC	*p* Value
DYNC1H1	2.521309	3.507417	0.986108	1.99E-23
PRKDC	1.734856	2.758504	1.023648	2.87E-20
CCDC88A	0.668268	1.238603	0.570335	7.28E-14
ADAMTS5	0.35781	0.533908	0.176098	0.024572407

*Abbreviation: LogFC* log fold change

Although that there was no significant difference in TMB between patients with high- and low-DEMG (Fig. 4G), low TMB was associated with better OS (Log-rank test, *p* < 0.001, Fig. 4H). When DEMG and TMB were e integrated to stratify the samples into TMB^high^/DEMG^low^, TMB^low^/DEMG^low^, TMB^high^/DEMG^high^, and TMB^low^/DEMG^high^ groups, significant differences were found among all groups (Fig. 4I, Log-rank test, *p* < 0.001), and patients in the TMB^low^/DEMG^low^ group exhibited the best OS.

### Validation of the risk signature in ICGC cohort

**Fig. 5:**
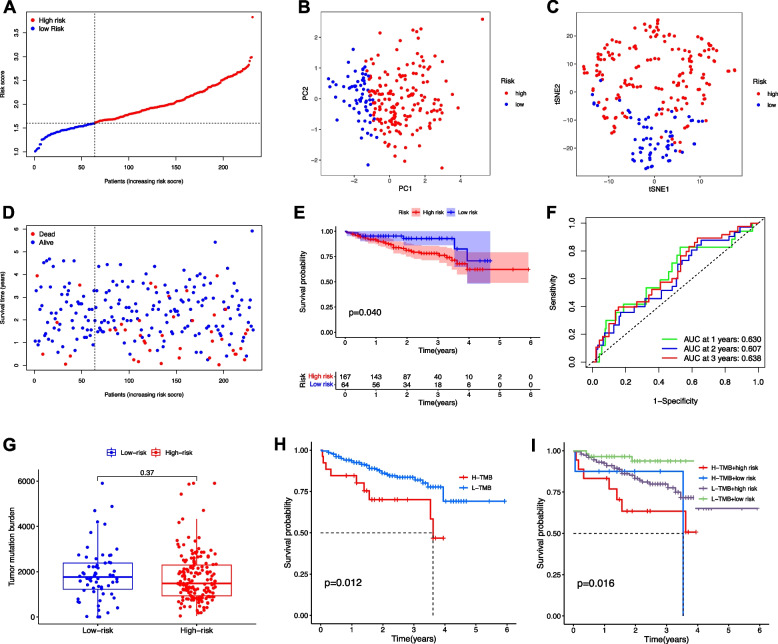
Validation of the risk score in the ICGC cohort. **A** Distribution and median value of the risk scores. **B** PCA plot and **C** t-SNE analysis. **D** Distributions of OS status, OS and risk score. **E** Kaplan-Meier curves for the OS of patients in the high-risk group and low-risk group. **F** AUC of time-dependent ROC curves confirmed the prognostic performance of the risk score. **G** Comparison of TMB between low- and high-risk groups. **H** Survival analysis based on the TMB. **I** Survival analysis for 4 groups by combining TMB and DEMG-based risk signature

To validate the robustness of the model constructed from TCGA cohort, a total of 273 HCC patients from ICGC cohort were utilized as the validation cohort. Based on the median risk score in TCGA cohort, the patients in ICGC cohort were also categorized into high- or low-risk groups (Fig. 5A). PCA and t-SNE analysis confirmed the discrete distribution of the patients in two subgroups (Fig. 5B-C). The survival outcomes of high-risk group were similar with those from TCGA cohort, Fig. 5D-E). ROC curve showed that this model had good predictive efficacy (AUC = 0.630, 0.607, and 0.638 for 1-, 3-, and 5-year survival, Fig. 5F). Although TMB is not significant between patients with DEMG^high^ and DEMG^low^ subgroups (Fig. 5G), TMB^low^ (Fig. 5H) and TMB^low^/DEMG^low^ (Fig. 5I) were also associated with good OS. These results collectively demonstrate that increased risk score was correlated with tumor progression.

### Independent prognostic value of the risk model

**Fig. 6:**
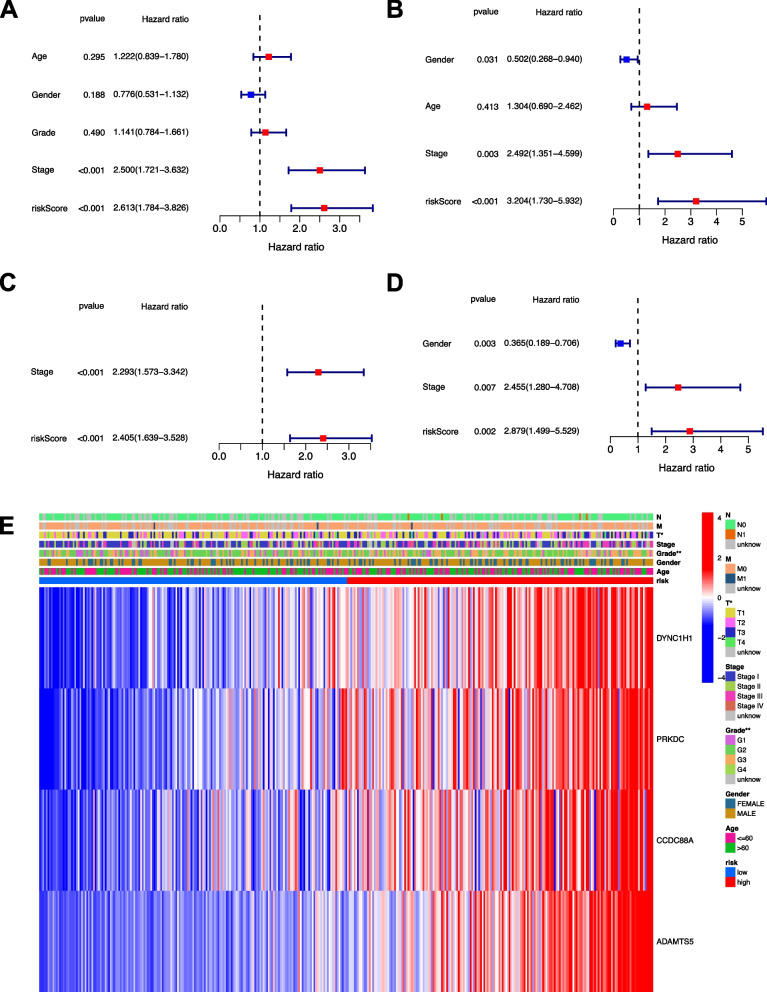
Univariate and multivariable Cox regression analyses of risk score. **A** Univariate and **B** multiple Cox regression analyses were performed in the TCGA cohort. **C** Univariate and **D** multiple Cox regression analyses were performed in the ICGC validation cohort. **E** Heatmap of clinical parameters for the TCGA cohort

The univariate Cox regression analysis suggested that the risk score was an independent prognostic factor (HR = 2.613, 95% CI: 1.784−3.826 for TCGA and HR: 3.204, 95% CI: 1.730−5.932 for ICGC, Fig. 6A-B). The multivariate Cox regression analysis also indicated that the risk score was a prognostic factor, after adjusting for other confounding factors (HR = 2.405, 95% CI: 1.639−3.528 for TCGA and HR: 2.879, 95% CI: 1.499−5.529 for ICGC, Fig. 6C-D). The clinical features of TCGA cohort suggested that grade and survival status of the patients were diversely distributed between low- and high-risk subgroups (Fig. 6E, *p*< 0.01).

### Functional analyses

**Fig. 7:**
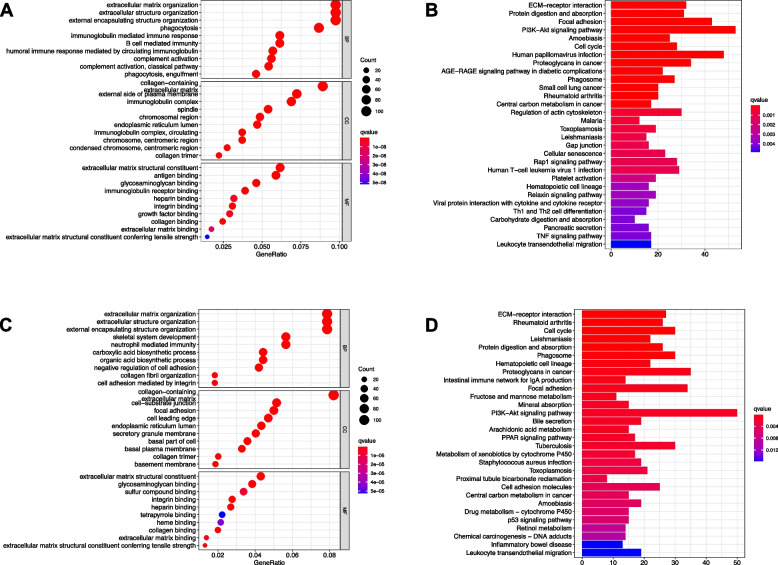
Functional analysis of the DGPM in TCGA and ICGC cohort. **A** Bubble graph for GO enrichment and **B** barplot graph for KEGG pathways in the TCGA cohort. **C** Bubble graph for GO enrichment and **D** barplot graph for KEGG pathways in the ICGC cohort

The DEGs were extracted by LASSO regression with “limma” R package using criteria FDR < 0.05 and |log2FC | ≥1 as indicated above. GO enrichment and KEGG pathway analysis were performed with these DEGs. DEGs in TCGA cohort were mainly correlated with the extracellular matrix, extracellular matrix (ECM)-receptor interaction, and PI3K-Akt signaling pathway (Fig. 7A-B). The biological processes, molecular functions and signaling pathways were validated by ICGC cohort (Fig. 7C-D).

### Comparison of the immune activity between subgroups

**Fig. 8:**
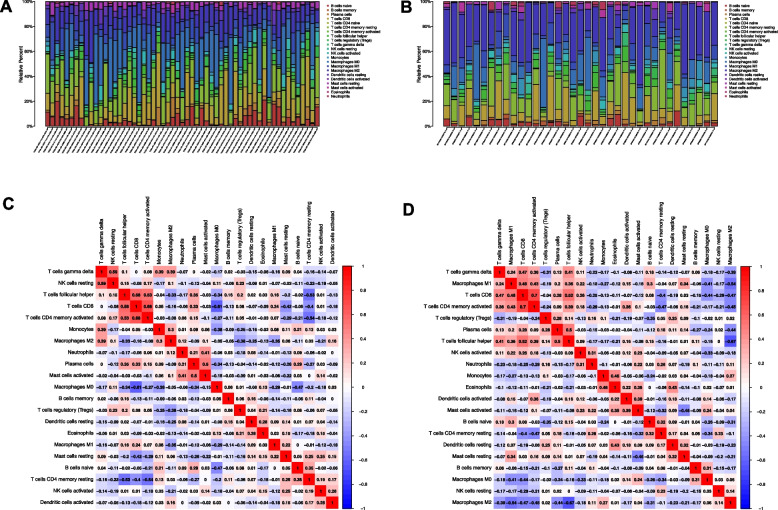
Immune cell infiltrations of TCGA and ICGC cohorts. Relative proportion of immune cell infiltration in (**A**) TCGA and (**B**) ICGC (**B**). Correlation analysis of immune cells in (**C**) TCGA and (**D**) ICGC

**Fig. 9.**
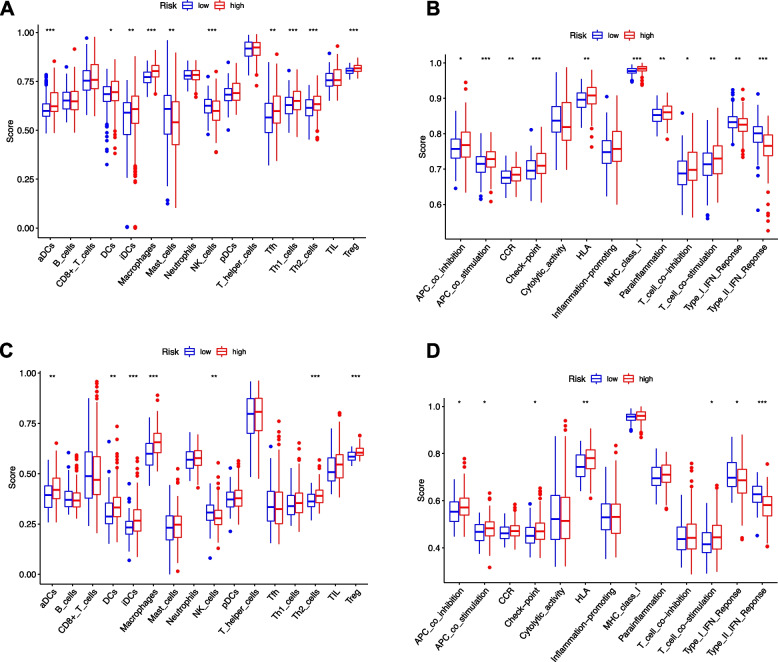
SsGSEA scores between different risk groups in TCGA and ICGC cohort. **A** The scores of 16 immune cells and **B** 13 immune-related functions in TCGA cohort are displayed in boxplots. **C** The scores of 16 immune cells and **D** 13 immune-related functions in ICGC cohort are displayed in boxplots. Adjusted *P* values were showed as: *, *p* < 0.05; **, *p* < 0.01; ***, *p* < 0.001

The ratio of immune cell infiltration and correlation of immune cells in TCGA and ICGC databases were shown in Fig. 8. The enrichment scores of 16 types of immune cells and the activity of 13 immune-related pathways were compared between low- and high-risk groups in both TCGA and ICGC cohorts by employing ssGSEA. In TCGA cohort (Fig. 9A), higher levels of infiltration of immune cells in high-risk subgroup, especially activated dendritic cells (aDCs), dendritic cells (DCs), immature dendritic cells (iDCs), macrophages, T helper (Th) cells (Tfh, Th1, and Th2 cells) and regulatory T (Treg) cells. Except for the type-1 and type-2 IFN response pathway, other immune pathways exhibited higher activity in the high-risk group in the TCGA cohort (Fig. 9B). When the immune status in ICGC cohort was evaluated, similar conclusions were drawn (Fig. 9C-D).

### Construction of nomogram with prognostic signature with clinical features

**Fig 10:**
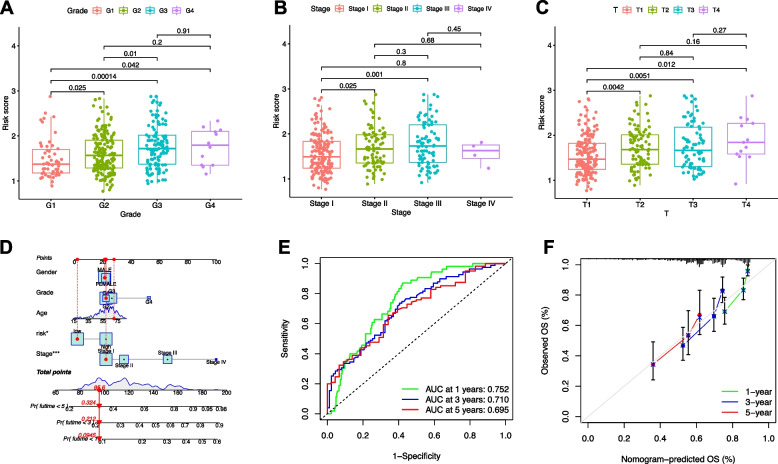
Correlation of DGPM with clinical features and construction of clinicopathological nomogram. **A** Correlation of risk score with **A** tumor grade, **B** clinical stage, and **C** T status. **D** Nomogram was constructed by grade, stage and risk signature for predicting survival. **E** AUCs for predicting 1-, 3-, and 5-year survival. **F** The 1-, 3-, and 5-year nomogram calibration curves

The risk score increased significantly with tumor grade, stage, and T category (Fig. 10A-C). A nomogram was constructed based on the prognostic signature and clinical parameters (Fig. 10D). The nomogram-predicted survival closely matched with the optimal predictive performance. The AUCs for the 1-, 3-, and 5-year were 0.752, 0.687, and 0.710, respectively (Fig. 10E). The nomogram had similar performance to that of an ideal model (Fig. 10F).

### PRKDC independently affected prognosis

**Fig. 11:**
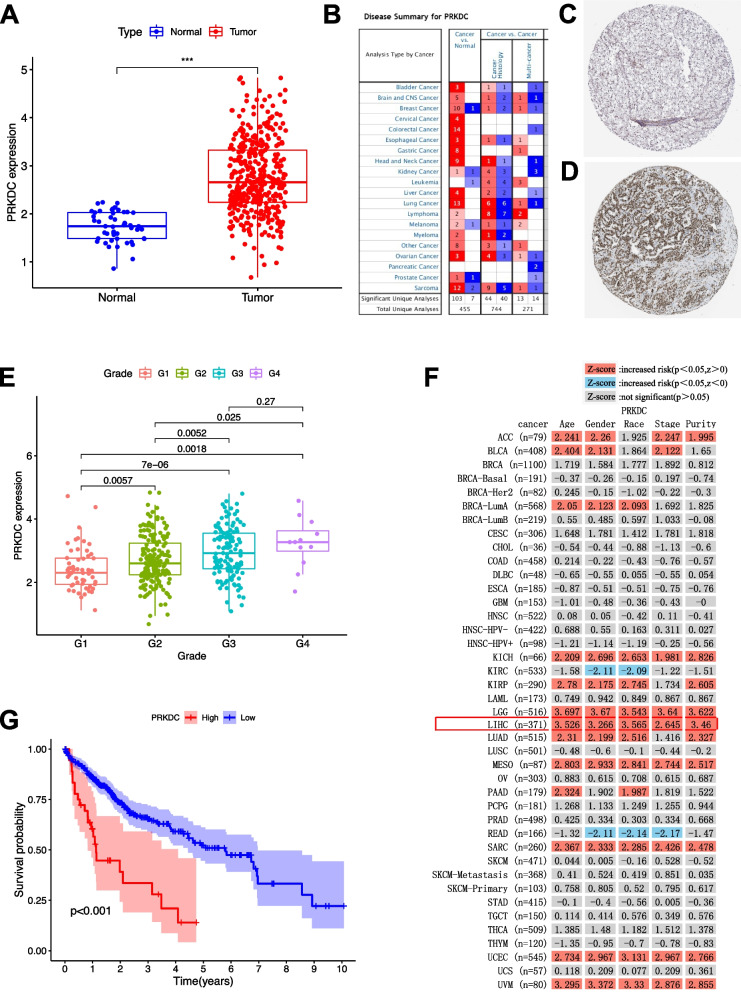
The clinical significance of PRKDC in HCC. **A** PRKDC are overexpressed in HCC tumor tissue. **B** Four analyses from ONCOMINE platform showing high expression of PRKDC. **C-D** Protein expression level of PRKDC was shown by The Human Protein Altas by immunohistochemistry. **E** Correlation of risk score with tumor grade. **F** The TIMER shows that the clinical outcome increased risk with the increase of PRKDC gene expression. **G** Lower PRKDC level predicts longer OS

PRKDC was only gene whose expression level was upregulated among the four prognostic-related DEMGs (DYNC1H1, PRKDC, CCDC88A, and ADAMTS5) (Log FC >1). PRKDC expression level was lower in adjacent normal specimens compared that of tumor tissues (Fig. 11A). There are 4 analyses showing high expression of PRKDC from ONCOMINE website (Fig. 11B). The protein expression level of included gene of signature was verified by The Human Protein Atlas (Fig. 11C-D). The results found that PRKDC expressed statistical significantly among different pathological grades (Fig. 11E, most *p* < 0.05). The TIMER shows that the clinical outcome of age, gender, race, stage, and purity increased risk with the increase of PRKDC gene expression (Fig. 11F). Kaplan-Meier analysis showed lower PRKDC expression level was correlated with longer OS time (Fig. 11G).

### PRKDC correlated with ICB key genes

**Fig. 12:**
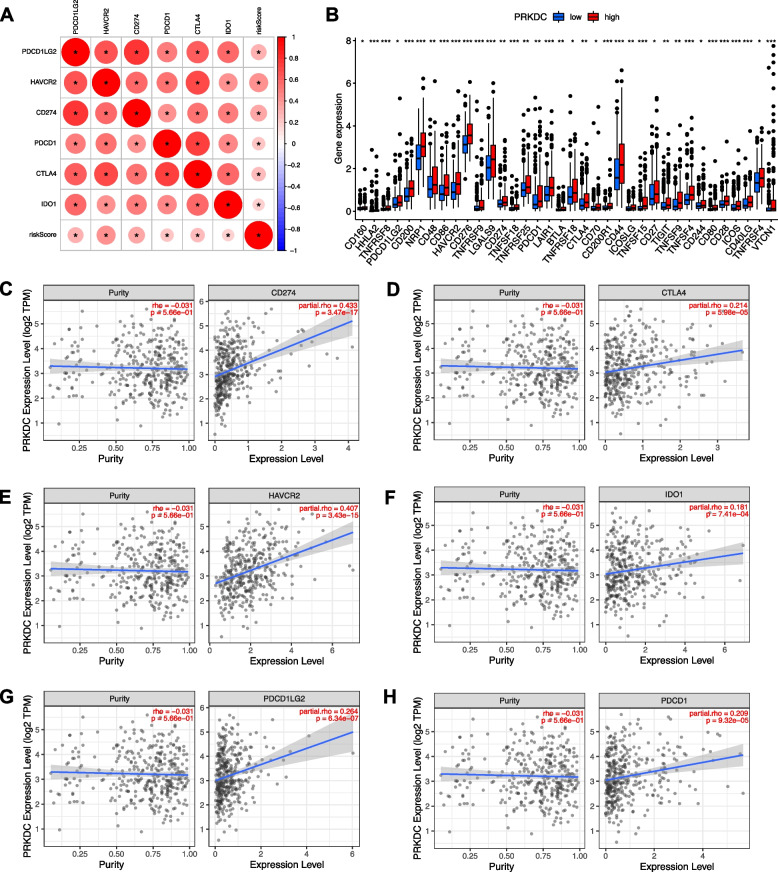
Association between PRKDC and immune checkpoint genes. **A** Correlation analysis between immune checkpoints CD274, PDCD1, PDCD1LG2, CTLA4, HAVCR2, and IDO1 and risk score. **B** Comparison of the expression levels of ICB-related genes between low- and high-PRKDC groups. Association between PRKDC and **C** CD274, **D** CTLA4, **E** HAVCR2, **F** IDO1, **G** PDCD1LG2, and **H** PDCD1

The correlation between six key ICB key targets and prognostic signature was analyzed to reveal the potential player of risk signature (Fig. 12A). Besides, expression levels of 36 out of 47 ICB-associated genes between low- and high-PRKDC groups were dysregulated in different subgroups (Fig. 12B). TIMER results adjusted by tumor purity showed PRKDC was positively correlated to CD274 (*r* = 0.433; *p *= 3.47e−17), CTLA4 (*r* = 0.214; *p *= 5.98e−05), HAVCR2 (*r* = 0.407; *p *= 3.43e−15), IDO1 (*r* = 0.181; *p* = 7.41e−04), PDCD1LG2 (*r* = 0.264; *p* = 6.34e−07) and PDCD1 (*r* = 0.209; *p *= 9.32e−05), suggesting PRKDC may exert a vital role in ICB treatment of HCC (Fig. 12C-H).

### Role of PRKDC in TME

**Fig. 13:**
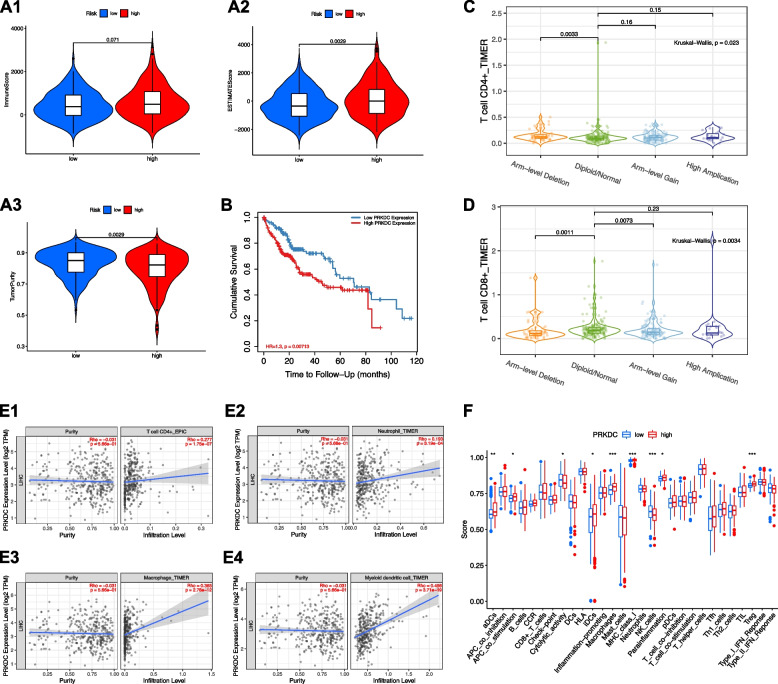
The role of PRKDC in TME features. **A** Comparison of (A1) immune score, (A2) ESTIMATE score and (A3) tumor purity between low- and high-PRKDC groups. **B** TIMER showed that low PRKDC was associated with good OS. Copy number of **C** CD4+ T-cells and **D** CD8+ T-cells in HCC. **E** Relationship between PRKDC with (E1) CD4+ T-cells, (E2) neutrophils, (E3) macrophages and (E4) myeloid dendritic cells. **F** Comparison of ssGSEA enrichment between low- and high-PRKDC groups.

HCC patients were classified into high- and low- PRKDC expression groups based on the median PRKDC expression level. ESTIMATE results suggested that patients with higher PRKDC expression had a higher stromal score, higher ESTIMATE score and lower tumor purity (Fig. 13A). TIMER showed that low PRKDC was associated with good OS (Fig. 13B). Arm-level deletion was the main type of mutation (Fig. 13C-D). PRKDC expression was positively correlated with immune cell infiltration (Fig. 13E). The results of ssGSEA suggested that except for cytolytic activity and NK cells, the infiltration fraction of aDCs, antigen-presentation cell (APC) co-inhibition, iDCs, macrophages, MHC-class-I, para-inflammation and Treg expression were significantly decreased when risk score is declining (Fig. 13F).

## Discussion

Exosome-derived mRNAs have gained attention due to their potential role in cancer development, progression, and as a source of diagnostic and therapeutic targets. Exosomes released by cancer cells can carry various molecular cargoes, including mRNA, contributing to the communication between cancer cells and their microenvironment. ExLR, mainly mRNAs are potential biomarkers in HCC [14]. Compared to tissue mRNAs, circulating emRNAs may reflect their original tissues and the relative fraction of immune cell types [15].

Exosome mRNA-related gene expression is associated with cancer development and progression. However, the characteristics and landscape of exosomal mRNAs are not fully understood. In this study, four exosomal mRNA-related genes were included to construct the prognostic model by univariate Cox and LASSO Cox regression analysis. Patients in high- and low-risk group had significantly different survival outcomes. Furthermore, ROC curve demonstrated that the prognostic signature was robust in predicting the OS for HCC patients. Additionally, when combined the prognostic signature with clinical parameters, the nomogram had satisfactory predictive performance for HCC. The prognostic signature correlated with tumor microenvironment and the expression of immune checkpoints. The flow diagram of our study is shown in supplementary Figure S1.

A better understanding of the biology represented by the selected genes can be obtained via analysis of functional networks or pathways that these genes based on their biological functions. The functional analysis indicated the prognostic signature was enriched in extracellular matrix, ECM-receptor interaction, and PI3K-Akt signaling pathway. Tumor cells release exosomes to interact with ECM. In turn, ECM regulates exosome secretion and uptake. The biomolecules of exosomes can impact ECM remodeling, which is associated with cancer progression. Tumor-derived exosomes are capable of modulating ECM and TME by disruption of cell adhesion formation and stimulation of the extracellular receptor signaling. Exosomes reach distal sites where they bind to cell surfaces and experience endocytosis with specific mechanisms [16]. ECM reorganization by exosomes contributes to physiological and pathologic angiogenesis. The surface to volume ratio of EVs is relatively large, which enables efficient surface interactions of EVs with cells and molecules. The surface interactions determine the fate of EVs by orchestra them to certain tissues or cell membrane. Focal adhesion pathway has also been implicated in KEGG pathway, which could regulate ECM. Being dynamic integrin-based adhesion complexes, focal adhesions anchor actin cytoskeleton to ECM, which transfers environmental stimuli to the cells and to change cell motility, adhesion, and shape [17]. Taken together, ECM modulation of the host tissue by tumor-derived exosome is involved in the crosstalk between cancer and the premetastatic niche [18].

Tumor-derived exosomes carry immunostimulatory and immunosuppressive receptor or ligands, partially mimicking the profiles of the parent cells [19]. Exosomes are involved in immune responses for tumorigenesis [20]. Exosomes-mediated signaling is contextual, and tumor-derived exosomes mainly mediate suppression in TME. The role of exosomes in antigen specific immune responses have also been demonstrated. In our study, the enriched immune cell types in high-risk group were aDCs, DCs, iDCs, Th2 cells, and Tregs. The results also found that most of the 13 immune-related functions were highly activated in the high-risk group, especially APC co-inhibition, APC co-stimulation, check-point, HLA, T cell co-stimulation. A large body of evidences supports the potential of tumor-derived exosomes to promote antigen-processing and differentiation capabilities of DC in TME. Exosomes carrying tumor-associated antigens (TAAs) and costimulatory molecules reprogram APCs, which could promote immune responses. The effects of tumor-derived exosomes on T-cell subsets are complex [19]. The proteins carried by exosomes can inhibit cytotoxicity and regulate immune-related genes in T cells [19]. Previous studies have reported tumor-derived exosomes could promote Treg activity and expansion [21, 22]. These results collectively indicated the risk signature was associated with TME.

Inhibitory receptors have been identified in cancers, including but not limited to PD-1, CTLA-4, LAG3, and TIM3, etc. [23],. Many biochemical studies have revealed complex and delicate regulation of checkpoint expression on cell surface [24]. Upon ligand engagement, checkpoints follow distinct signaling mechanisms to inhibit antitumor immunity. Exosomes communicate between tumor cells of immune cells and stromal cells by transferring message, contributing to immune escape. For example, tumor-derived exosomes containing PD-L1 can mimic the function of PD-L1 on cell surface. The association between levels of circulating exosomal PD-L1 and response to anti-PD-1/PD-L1 antibody therapy has been documented [25]. The correlation of the prognostic signature of exosome mRNA-related genes with ICB key targets reveal the risk signature has a potential role in the ICB treatment of HCC.

There are several proposed exosome-mediated drug resistance mechanisms, including exosome-mediated transfer of miRNAs, neutralization of antibody-based drugs, and drug export via the exosome pathway [26]. Exosomes could also induce therapy-resistance by promoting anti-apoptotic pathways and alteration of signaling transduction [27]. The PI3K-Akt pathway has been linked to modulate the multidrug resistance of various cancers [28]. For example, the exosome-mediated PI3k/Akt/mTOR signaling pathway has been implicated in cervical cancer [29]. Similarly, gastric cancer-derived exosomes facilitate the proliferation of recipient cell via PI3K/Akt signaling pathway [30]. However, the role of exosome mRNA-related gene signature in the drug sensitivity prediction needs further validation in larger clinical samples. More studies are required to fully uncover the complexities of exosome-derived mRNA in cancer. Ongoing research aims to unravel the specific mRNA cargoes carried by exosomes, their functional implications, and their potential as therapeutic targets or tools for cancer management.

Among the four genes selected to construct the prognostic signature, PRKDC was recently identified as a new biomarker and potential target for immunotherapy [31]. PRKDC gene encodes catalytic subunit of a nuclear DNA-dependent serine/threonine protein kinase (DNA-PKcs) [32]. As a catalytic protein, DNA-PKcs together with Ku70 and Ku80 constitute a DNA-dependent protein kinase (DNA-PK) [33]. The degradation of DNA-PK is regulated by vasolin-containing protein (VCP), which is found in the exosomes from gliomas [34]. DNA-PK is involved in DNA double-strand break repair, immunocompetence, and genomic integrity [35]. Previous studies indicated DNA-PK is a candidate driver of hepatocarcinogenesis which can predict treatment response and patient survival [36]. DNA-PK has emerged as a potential therapeutic target in various cancer types. By inhibition of its kinase function, DNA-PK inhibitors could potentiate DNA damage [37]. The investigation of DNA-PK inhibitor employs with monotherapy and combination strategy [38].

Our study is the first to construct and validate an exosome mRNA-related gene risk model based on four exosome-related genes, which can serve as an independent prognostic factor in HCC patients. However, our study has some limitations. First, the validation of the prognostic signature was performed in public database, larger clinical cohorts are need to confirm the value of this signature. Second, the risk model was solely established on exosome mRNA-related genes, the information of lncRNA, miRNA, and circRNA were not included in our study. Third, the relationship of this signature with tumor microenvironment is preliminary and hypothesis-generating, therefore, experimental confirmation is required in the future.

## Conclusions

In summary, an exosome mRNA-related prognostic risk model was established and validated to predict the prognosis of HCC patients and associated with immune infiltration. The risk model can serve as an independent prognostic factor for HCC patients and highlights the functional significance of mRNAs in exosomes.

### Supplementary Information

**Supplementary Material 1.**

**Supplementary Material 2.**

## Data Availability

Publicly available datasets from TCGA (https://portal.gdc.cancer.gov/), ICGC (https://dcc.icgc.org/) and exoRBase (http://exorbase.org/exoRBaseV2/download/toIndex) were analyzed in this study. were analyzed in this study.
