# Supplementary material for: An exosome mRNA-related gene risk model to evaluate the tumor microenvironment and predict prognosis in hepatocellular carcinoma
Source: BMC Med Genomics. 2024 Apr 16;17:86. doi: 10.1186/s12920-024-01865-z (PMC11020893; doi:10.1186/s12920-024-01865-z)
Supplement: Supplementary file 1 — Supplementary Material 1. [file 12920_2024_1865_MOESM1_ESM.pdf]

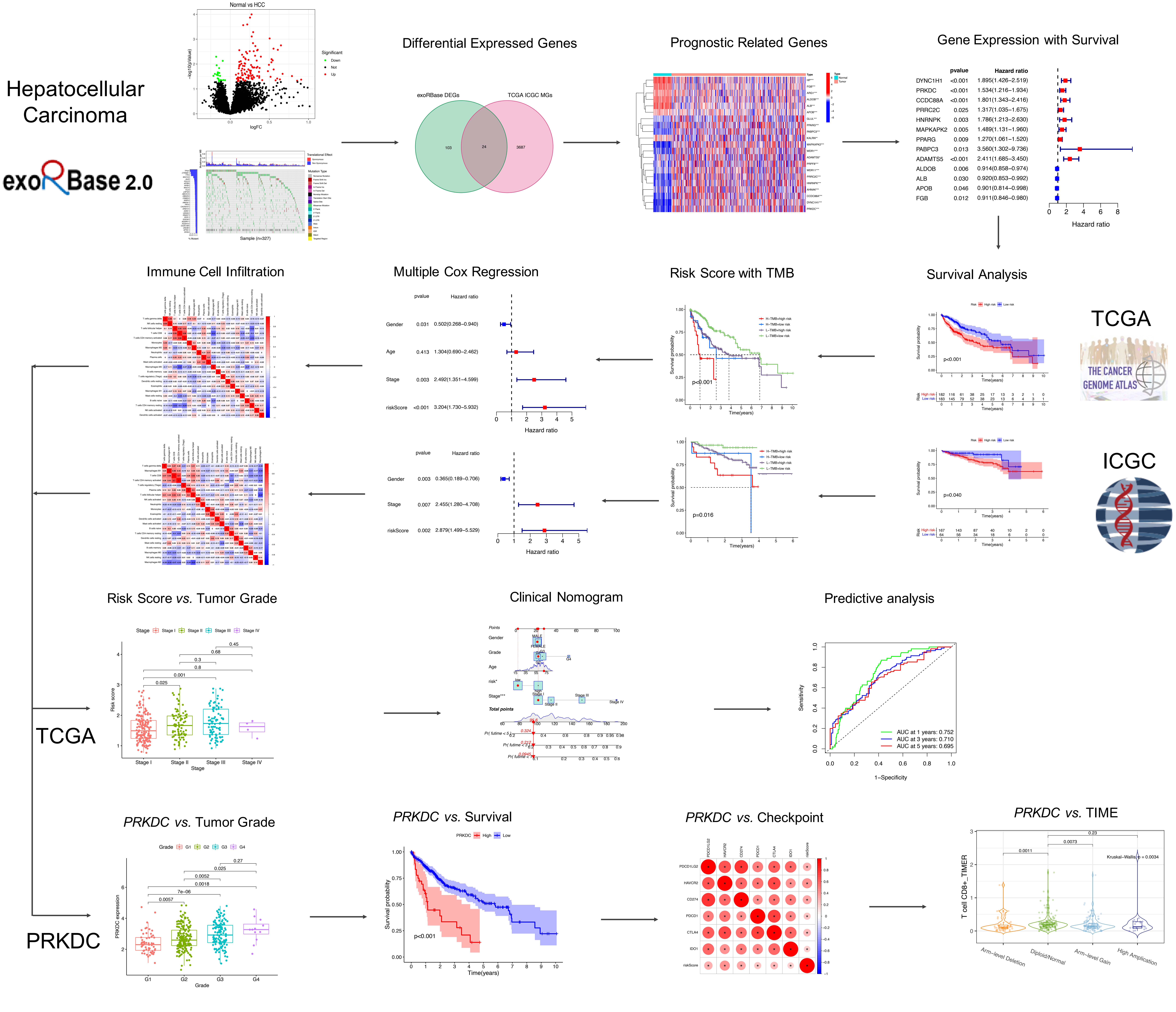

Supplementary Figure S1

Flow chart of the study.
