# Supplementary material for: An exosome mRNA-related gene risk model to evaluate the tumor microenvironment and predict prognosis in hepatocellular carcinoma
Source: BMC Med Genomics. 2024 Apr 16;17:86. doi: 10.1186/s12920-024-01865-z (PMC11020893; doi:10.1186/s12920-024-01865-z)
Supplement: Supplementary file 2 — Supplementary Material 2. [file 12920_2024_1865_MOESM2_ESM.docx]

# Supplementary Tables

## Supplementary Table S1. Normal vs. benign of differentially expressed genes.

| Gene | Control Mean | Treatment Mean | LogFC | p Value |
| --- | --- | --- | --- | --- |
| MMP9 | 18.0951354 | 9.84019731 | -0.8788428 | 0.01150881 |
| MMP14 | 8.3477517 | 4.65239363 | -0.8434145 | 0.0172903 |
| CHI3L1 | 7.67342996 | 4.37441592 | -0.8107812 | 0.02367589 |
| NACA2 | 14.5795364 | 10.1071428 | -0.5285696 | 0.03042092 |
| EIF2S3B | 5.25452112 | 3.94422612 | -0.413817 | 0.01597421 |
| PABPC3 | 8.61197559 | 6.69705216 | -0.362818 | 0.00174063 |
| POTEE | 4.51180104 | 3.57217761 | -0.3368996 | 0.01576345 |
| HIST1H1D | 851.510508 | 683.828281 | -0.3163902 | 0.00109885 |
| TBX21 | 161.013283 | 132.23889 | -0.2840332 | 0.00366403 |
| SCMH1 | 74.574339 | 62.7984532 | -0.2479503 | 0.00510932 |
| PTPN22 | 101.005219 | 85.511342 | -0.2402421 | 0.00499881 |
| HIST1H1E | 2245.35382 | 1907.95405 | -0.2349164 | 0.00284367 |
| VEGFB | 155.71618 | 132.484597 | -0.2330942 | 0.0281254 |
| SAMD3 | 62.3544671 | 53.3466474 | -0.2250953 | 0.00985979 |
| HIST1H1C | 2188.64874 | 1878.79681 | -0.2202314 | 0.00282976 |
| HIST1H3I | 838.707882 | 722.006198 | -0.2161572 | 0.02426863 |
| CISH | 54.9046498 | 47.3718511 | -0.2128983 | 0.03078518 |
| SIRT2 | 27.0545401 | 23.408648 | -0.2088291 | 0.02684559 |
| NME4 | 58.2390383 | 50.394026 | -0.2087338 | 0.04145394 |
| METTL25 | 35.1183239 | 30.4322039 | -0.2066252 | 0.04852657 |
| FKBP3 | 129.445726 | 113.21241 | -0.1933152 | 0.02154356 |
| ID2 | 130.333896 | 114.926994 | -0.1814946 | 0.03045683 |
| PHC3 | 122.246121 | 114.980804 | -0.0883957 | 0.04394308 |
| RARS | 92.9515343 | 83.0261313 | -0.1629132 | 0.02732361 |
| YBX1 | 3842.39705 | 3441.87287 | -0.1588128 | 0.0160543 |
| HIST1H2BK | 4011.00216 | 3603.58521 | -0.1545298 | 0.00849664 |
| ATP5F1C | 234.202334 | 210.902985 | -0.1511759 | 0.03992267 |
| PNRC1 | 1102.92517 | 995.254514 | -0.1481975 | 0.03698124 |
| CDC42EP3 | 50.3791888 | 45.5942535 | -0.1439759 | 0.02871279 |
| ABHD17A | 85.0194754 | 77.2920534 | -0.1374733 | 0.04286847 |
| HIST1H2BC | 2135.06408 | 1943.04326 | -0.1359613 | 0.03244708 |
| ESYT2 | 125.323152 | 114.329123 | -0.13246 | 0.03562794 |
| EIF1 | 632.947709 | 578.559729 | -0.1296204 | 0.00035547 |
| AES | 347.527776 | 319.377584 | -0.1218652 | 0.04780125 |
| RPL15 | 730.064952 | 671.451459 | -0.1207417 | 0.03087589 |
| ENSA | 82.009818 | 75.7579076 | -0.1144002 | 0.03898263 |
| RALY | 112.571449 | 104.411476 | -0.1085607 | 0.03348748 |
| RPLP1 | 2248.21951 | 2085.51753 | -0.1083775 | 0.04264066 |
| TBC1D1 | 96.001125 | 89.2684368 | -0.1049011 | 0.02965628 |
| SETD3 | 183.800685 | 171.051023 | -0.1037154 | 0.03913178 |
| PCBP1 | 834.581414 | 777.706057 | -0.1018278 | 0.02414297 |
| HIPK3 | 289.575978 | 272.281964 | -0.0888405 | 0.00811654 |
| STAG2 | 146.398328 | 155.786718 | 0.08967316 | 0.046336 |
| RAB8A | 314.313461 | 335.47893 | 0.0940181 | 0.04982434 |
| TAOK3 | 99.2689867 | 106.237365 | 0.0978763 | 0.02035097 |
| STX7 | 38.9936482 | 41.7859445 | 0.09977861 | 0.04755756 |
| ISCU | 156.896723 | 169.894811 | 0.11482657 | 0.00816563 |
| THOC7 | 116.539964 | 129.269749 | 0.14955994 | 0.02293413 |
| WDR11 | 35.8271188 | 38.8108236 | 0.11540702 | 0.03672599 |
| HNRNPK | 629.605709 | 682.879297 | 0.11718198 | 1.04E-05 |
| MYL6 | 631.363323 | 678.190055 | 0.10321918 | 0.04230981 |
| HMGB1 | 264.247917 | 287.875329 | 0.12355206 | 0.01938179 |
| AP1S2 | 169.061274 | 184.347231 | 0.12487952 | 0.01772964 |
| MEF2C | 73.7021255 | 80.3991838 | 0.12547463 | 0.00415967 |
| ARL8B | 114.277084 | 124.724833 | 0.12621261 | 0.02101515 |
| PHIP | 38.2725245 | 41.7755009 | 0.12634806 | 0.02893476 |
| TMEM167A | 106.220389 | 116.713437 | 0.13590994 | 0.00958968 |
| SECISBP2 | 33.0807666 | 36.3778283 | 0.13706675 | 0.03554185 |
| SHC1 | 39.8760951 | 45.197951 | 0.18073323 | 0.01683612 |
| VDAC1 | 100.877992 | 111.283497 | 0.14162819 | 0.02247882 |
| WASHC4 | 48.9232992 | 54.0527431 | 0.14384614 | 0.03353709 |
| VPS41 | 58.3555353 | 64.7142403 | 0.1492137 | 0.00479232 |
| EIF4H | 226.443761 | 251.191097 | 0.14963254 | 0.0148241 |
| SRSF11 | 51.5568261 | 57.22168 | 0.1503984 | 0.04997957 |
| UBR4 | 27.8069544 | 31.0176737 | 0.15764475 | 0.01938891 |
| LPP | 20.8455306 | 23.2557087 | 0.15784681 | 0.01207018 |
| PPP2CA | 64.1343082 | 71.5804519 | 0.15846933 | 0.02248106 |
| ELF1 | 369.337191 | 412.955167 | 0.16104662 | 0.00267198 |
| KIAA0513 | 43.7969212 | 49.068799 | 0.1639765 | 0.03661649 |
| LNPEP | 20.1198932 | 22.5683324 | 0.16567718 | 0.04869658 |
| SRSF6 | 171.316558 | 192.540988 | 0.168501 | 0.04578218 |
| HNRNPH1 | 94.9738641 | 107.090589 | 0.17322924 | 0.00825658 |
| GTF2B | 84.9483377 | 96.1591075 | 0.17883779 | 0.03872916 |
| ATP5F1E | 228.295108 | 259.137124 | 0.18281577 | 0.02498197 |
| PRKAR1A | 189.46241 | 215.38898 | 0.1850328 | 0.00014344 |
| TNFAIP8 | 42.0965872 | 48.0104999 | 0.18964668 | 0.0143359 |
| FNBP1L | 33.9931842 | 38.8043025 | 0.19097112 | 0.03689424 |
| RAB4A | 105.173879 | 120.065433 | 0.19104442 | 0.03938692 |
| UBA7 | 44.5846298 | 50.9576155 | 0.19275133 | 0.01618428 |
| GPD2 | 28.4778417 | 32.7112057 | 0.19994513 | 0.04224782 |
| SAT1 | 939.780551 | 1092.78954 | 0.21761977 | 0.02957889 |
| NRIP1 | 58.8460325 | 68.6411805 | 0.22212922 | 0.03268256 |
| POMP | 162.04745 | 191.164049 | 0.23839491 | 0.00656939 |
| CLK4 | 8.82581648 | 10.4289344 | 0.24079011 | 0.04807284 |
| ROMO1 | 94.3644439 | 112.023094 | 0.24748091 | 0.02865359 |
| ENY2 | 17.4347117 | 20.8372282 | 0.25720087 | 0.02394843 |
| RDH11 | 43.9049644 | 52.6794207 | 0.2628554 | 0.04807865 |
| HBA2 | 7506.26003 | 9101.61804 | 0.27802877 | 0.04758839 |
| NKTR | 15.6398514 | 19.1624747 | 0.29305708 | 0.02550547 |
| RPS29 | 100.197009 | 124.089489 | 0.30854147 | 0.01076988 |
| MINOS1 | 4.23818785 | 5.37548552 | 0.34294753 | 0.04686493 |
| SZT2 | 4.77459804 | 6.12120066 | 0.35843538 | 0.0037577 |
| RPL26 | 101.926243 | 135.408526 | 0.40979303 | 0.01981681 |
| PCDH9 | 1.13847919 | 1.52321689 | 0.42001345 | 0.01411178 |
| RRNAD1 | 4.05522349 | 5.46283008 | 0.42986712 | 0.03835961 |
| NDUFA11 | 3.75222945 | 5.09245533 | 0.44061337 | 0.00997791 |
| EIF5AL1 | 1.90717736 | 2.68628798 | 0.49417496 | 0.03521026 |
| DEFA3 | 42.4581002 | 64.570995 | 0.60484644 | 0.03521442 |
| LDHB | 7.36821503 | 11.3126281 | 0.61854706 | 0.00705519 |
| AL136454.1 | 34.9673652 | 56.290373 | 0.68687912 | 0.00353648 |
| PET100 | 4.23011409 | 6.91296365 | 0.70860776 | 0.00523591 |

## Supplementary Table S2. Normal vs. HCC of differentially expressed genes.

| Gene | Control Mean | Treatment Mean | LogFC | p Value |
| --- | --- | --- | --- | --- |
| SZT2 | 6.08072349 | 4.9396786 | -0.2998258 | 0.02178831 |
| OSBPL10 | 11.0354848 | 9.39472373 | -0.2322274 | 0.03140359 |
| LETMD1 | 16.7575735 | 14.3853783 | -0.2202101 | 0.04818477 |
| HLA-DQA1 | 35.7278289 | 30.9482575 | -0.2071901 | 0.02531924 |
| MS4A1 | 59.1461737 | 51.4510324 | -0.2010848 | 0.03684373 |
| WDFY4 | 32.3382429 | 28.3103922 | -0.1919096 | 0.01648894 |
| AHNAK | 359.369174 | 315.544021 | -0.1876254 | 0.04399864 |
| BANK1 | 154.63511 | 137.187389 | -0.1727201 | 0.00380593 |
| HLA-DRA | 1271.18903 | 1129.80278 | -0.1701076 | 0.01473885 |
| RUNX1 | 38.2692149 | 34.1014865 | -0.1663497 | 0.01544829 |
| RAB30 | 19.4573329 | 17.4296565 | -0.1587698 | 0.02359855 |
| WDR11 | 37.9325959 | 34.1956006 | -0.1496274 | 0.00871686 |
| RCAN3 | 80.3103078 | 72.5018664 | -0.147567 | 0.02572933 |
| DYNC1H1 | 30.0374197 | 27.2059323 | -0.1428396 | 0.03746347 |
| PRKDC | 44.0919043 | 40.0185209 | -0.1398459 | 0.0171382 |
| CCDC88A | 13.6674899 | 12.4726625 | -0.1319788 | 0.04104186 |
| PRPF8 | 97.5157264 | 90.0010073 | -0.1156938 | 0.02979054 |
| PHIP | 41.539084 | 38.4550351 | -0.1112969 | 0.04997455 |
| CELF2 | 78.7380743 | 73.1574051 | -0.1060575 | 0.02484998 |
| MEF2C | 80.4686246 | 74.9710829 | -0.1020921 | 0.01441537 |
| PRRC2C | 179.235375 | 167.336516 | -0.0991031 | 0.01277039 |
| NCOA3 | 110.70236 | 103.734869 | -0.0937851 | 0.03936053 |
| CLINT1 | 109.685625 | 103.538531 | -0.0832067 | 0.04039756 |
| PHC3 | 118.344892 | 112.345229 | -0.0750586 | 0.04191205 |
| STK4 | 222.052469 | 212.47819 | -0.0635859 | 0.04193118 |
| HNRNPK | 693.562577 | 664.215638 | -0.0623744 | 0.01416602 |
| ISCU | 163.854543 | 174.437887 | 0.09029775 | 0.04449356 |
| OTUD5 | 78.3521616 | 83.5610465 | 0.09285748 | 0.04973161 |
| GLUL | 143.441449 | 153.283583 | 0.09574123 | 0.02056101 |
| WDR1 | 347.436887 | 374.153603 | 0.10687974 | 0.03833627 |
| MAPKAPK2 | 138.929796 | 149.845647 | 0.10912113 | 0.01269501 |
| EDF1 | 491.406669 | 533.180202 | 0.11770577 | 0.0438358 |
| ARHGEF12 | 89.5919469 | 97.2091783 | 0.11772348 | 0.04076702 |
| NAB1 | 69.9655256 | 76.2225655 | 0.12357394 | 0.03491683 |
| PARK7 | 248.980389 | 271.57828 | 0.12533599 | 0.04863071 |
| GABARAPL2 | 373.129515 | 408.632212 | 0.13112645 | 0.04432229 |
| PCMT1 | 83.1057694 | 91.0172721 | 0.13119171 | 0.04907828 |
| CASP3 | 74.2083024 | 81.3975961 | 0.13340558 | 0.04548196 |
| UBE2Q1 | 108.125949 | 118.727955 | 0.13494687 | 0.00489698 |
| G6PD | 89.4267854 | 98.2881133 | 0.13630994 | 0.03028613 |
| MYL6 | 616.803485 | 678.577254 | 0.13770216 | 0.00794358 |
| RAB11B | 164.020458 | 181.069002 | 0.14266382 | 0.00375272 |
| YES1 | 55.4112242 | 61.2499626 | 0.14453072 | 0.03679379 |
| VPS37B | 32.5210425 | 35.9702552 | 0.14543089 | 0.01165359 |
| THOC7 | 118.781415 | 131.932896 | 0.15149521 | 0.04508414 |
| ETS2 | 90.5492126 | 100.604414 | 0.1519196 | 0.04293615 |
| ATP6V1F | 361.370186 | 402.68254 | 0.15616543 | 0.03640374 |
| MTRF1L | 53.8259282 | 60.094859 | 0.15894028 | 0.03260835 |
| NUTF2 | 87.9516415 | 98.1999745 | 0.15901215 | 0.04519829 |
| IL32 | 171.84691 | 192.196546 | 0.1614585 | 0.03779411 |
| SHC1 | 41.686934 | 46.6388514 | 0.16193699 | 0.02617263 |
| SMYD2 | 24.6463536 | 27.6778279 | 0.16735651 | 0.02977954 |
| GYPC | 262.575826 | 297.674214 | 0.18100015 | 0.03541178 |
| PGD | 295.784601 | 335.417518 | 0.18141109 | 0.01302879 |
| NME4 | 49.1638624 | 56.0191186 | 0.18832102 | 0.04065799 |
| KALRN | 6.1384464 | 7.02422675 | 0.19446585 | 0.04931932 |
| KIF1C | 209.195362 | 241.400694 | 0.20657896 | 0.02774766 |
| HNRNPA1P48 | 19.5849464 | 22.6099015 | 0.20720953 | 0.04376717 |
| NES | 74.1982318 | 86.1104132 | 0.21480291 | 0.02488731 |
| RAB32 | 209.691789 | 244.045723 | 0.2188811 | 0.0113358 |
| CYB5A | 8.82497847 | 10.2831972 | 0.22062423 | 0.03972648 |
| RAB13 | 30.230519 | 35.3287941 | 0.22483876 | 0.04896626 |
| VASH1 | 66.2068263 | 77.7282374 | 0.23145883 | 0.02669792 |
| NAMPT | 131.942399 | 155.737151 | 0.2392049 | 0.01957836 |
| PPARG | 10.8714567 | 12.9179072 | 0.24882709 | 0.04872775 |
| GCA | 90.727798 | 107.868023 | 0.2496507 | 0.03368663 |
| ELL2 | 15.6170604 | 18.6736849 | 0.25788372 | 0.02763815 |
| RGS2 | 208.477903 | 251.882014 | 0.27285363 | 0.02395643 |
| S100A11 | 3358.71766 | 4079.10341 | 0.28034156 | 0.01217531 |
| SH3PXD2A | 40.2784204 | 49.1086823 | 0.28597101 | 0.00302422 |
| DAP | 281.978723 | 344.950137 | 0.29080153 | 0.00917404 |
| CBWD6 | 1.55298006 | 1.91598664 | 0.30304819 | 0.04115165 |
| SLC25A4 | 5.1037668 | 6.38169594 | 0.32237746 | 0.04638489 |
| MYL9 | 668.444776 | 838.611513 | 0.32719426 | 0.03653338 |
| AKAP5 | 4.94396929 | 6.2275943 | 0.33300518 | 0.02478394 |
| CYSTM1 | 17.3699055 | 21.8961756 | 0.33408901 | 0.00885108 |
| RPS4Y1 | 312.466676 | 394.626883 | 0.3367869 | 0.01675105 |
| POTEE | 3.53128182 | 4.4764818 | 0.34217336 | 0.0197909 |
| SDC3 | 4.01279952 | 5.12629041 | 0.35330613 | 0.03232783 |
| S100A9 | 4057.00472 | 5186.61056 | 0.35437707 | 0.00538963 |
| LRRC4 | 4.68871918 | 5.99497687 | 0.35456031 | 0.02681841 |
| USP9Y | 9.00679895 | 11.52178 | 0.35527726 | 0.02484483 |
| HIST1H2AJ | 845.889463 | 1089.77127 | 0.36548431 | 0.02862704 |
| S100A12 | 256.719238 | 331.082243 | 0.36699823 | 0.04733012 |
| MRO | 7.22981262 | 9.43927224 | 0.38471738 | 0.04604116 |
| MTRNR2L12 | 7358.75058 | 9615.41743 | 0.38588865 | 0.04475793 |
| MANSC1 | 3.08199455 | 4.02837368 | 0.38633321 | 0.04408897 |
| EIF2S3B | 3.75274279 | 4.94096128 | 0.39684634 | 0.01034245 |
| KRT23 | 10.4063852 | 13.7078295 | 0.39753114 | 0.03911488 |
| ZFY | 22.7606507 | 29.9856966 | 0.39773269 | 0.01029165 |
| PROK2 | 38.0412791 | 50.1316111 | 0.39815484 | 0.0061479 |
| SETSIP | 23.8898953 | 31.4827003 | 0.39815876 | 0.00622963 |
| EIF1AY | 49.7167371 | 66.2721461 | 0.41467102 | 0.0139697 |
| PABPC3 | 6.88850358 | 9.19447046 | 0.41657587 | 0.0006553 |
| TGFB3 | 3.96435961 | 5.34533574 | 0.43119273 | 0.04136545 |
| NACA2 | 8.93770913 | 12.1467532 | 0.44259374 | 0.02698211 |
| APOC1 | 20.5166632 | 28.0736872 | 0.45242244 | 0.02727499 |
| EIF3CL | 20.636827 | 28.4795713 | 0.46470626 | 0.00156688 |
| MTRNR2L3 | 4.78985244 | 6.65497689 | 0.47445245 | 0.03438206 |
| ARG1 | 17.0798992 | 23.8307631 | 0.48052569 | 0.01135931 |
| TLCD2 | 2.85909019 | 3.99979272 | 0.48436911 | 0.01318436 |
| MTRNR2L8 | 12894.4384 | 18070.9305 | 0.48692186 | 0.00386125 |
| UQCRHL | 16.3115245 | 22.875787 | 0.48792975 | 0.00024934 |
| APOE | 22.8257134 | 32.7456881 | 0.520645 | 0.0213906 |
| AC011195.2 | 1.97694336 | 2.86958016 | 0.53756814 | 0.02884371 |
| RAB6D | 3.30084327 | 4.81895816 | 0.54588664 | 5.89E-05 |
| ADAMTS5 | 2.96596634 | 4.33959065 | 0.54905674 | 0.00887505 |
| HIST2H4B | 5.88002635 | 8.61058927 | 0.55028935 | 0.00070245 |
| H2BFS | 8.30505172 | 12.2956853 | 0.56609109 | 0.006102 |
| MMP9 | 4.06247211 | 6.04144908 | 0.57253672 | 0.04657702 |
| HIST2H2AA3 | 55.7116134 | 83.0921913 | 0.57673481 | 6.12E-05 |
| POTEG | 0.92678109 | 1.4184274 | 0.61399179 | 0.00358493 |
| HIST2H2AA4 | 12.7973293 | 19.9044802 | 0.63725044 | 0.01997023 |
| MTRNR2L4 | 1.48161469 | 2.3053411 | 0.63780992 | 0.00825402 |
| HIST2H3C | 1.82317008 | 2.90587094 | 0.67252148 | 0.02302272 |
| ALDOB | 3.09404931 | 4.94599192 | 0.67676369 | 0.01397557 |
| RERG | 1.56528216 | 2.53572209 | 0.6959739 | 0.03870484 |
| TTR | 3.44424679 | 5.91646938 | 0.78054799 | 0.00533578 |
| ANGPTL3 | 1.54441194 | 2.82859448 | 0.87302775 | 0.0339429 |
| CYP2E1 | 2.01833509 | 3.72350796 | 0.88349673 | 0.00559604 |
| ALB | 117.149181 | 219.667799 | 0.90697653 | 0.01681827 |
| APOA1 | 2.75004392 | 5.1665705 | 0.9097523 | 0.01231167 |
| RBP4 | 2.53494981 | 4.94722041 | 0.96466099 | 0.02740933 |
| APOC3 | 8.46877493 | 16.5833291 | 0.96950846 | 0.02086419 |
| VTN | 3.59354845 | 7.1972627 | 1.00203918 | 0.01203831 |
| APOB | 1.33800712 | 2.6896434 | 1.00732911 | 0.00943907 |
| HP | 2.14976926 | 4.50935755 | 1.06874009 | 0.01416368 |
| ORM1 | 13.8143475 | 29.0930571 | 1.07450749 | 0.0024445 |
| ITIH2 | 1.70405067 | 3.65134251 | 1.09945878 | 0.04202512 |
| APOH | 14.1053059 | 30.9115308 | 1.13190715 | 0.04246751 |
| APOA2 | 9.33912622 | 20.4689398 | 1.1320769 | 0.00309333 |
| FGB | 8.59964052 | 20.4925156 | 1.25274884 | 0.0245207 |
